# Current News and Opinion

**Published:** 1886-03

**Authors:** 


### NINTH INTERNATIONAL MEDICAL CONGRESS.

### SECTION ON DENTAL AND ORAL SURGERY.

As there seems to be some misapprehension in the minds of some and earnest enquiry by others as to the status of the Section on Dental and Oral Surgery in the International Medical Congress to be held in Washington, D. C., in 1887, it seems right and proper that some statement be now made in regard to the organization and progress of the work.

It is very generally known that the Section has been established and organized. The following officers have been appointed, viz: a president, one vice-president and two secretaries.

Fourteen gentlemen of recognized ability and high professional standing from various parts of the country have accepted position upon the Council, and have pledged themselves to do all they can to make this Section a success. At the next meeting of the Executive Committee ten or twelve more names will be added to the Council, as may seem best. Much of the preliminary work in arranging the matters of the Section has been in the main accomplished.

A programme embracing the subjects of greatest interest to the profession of the world has been outlined and is now under consideration, and as soon as completed the secretaries will open correspondence with the eminent men of the profession in Europe and America relative to the work to be done. Quite a number have already indicated a desire to prepare papers, or at least to take some part in the work.

We not only expect but are assured that this Section will receive the hearty support and co-operation of dental specialists, both at home and abroad, and with such manifestations great hope is entertained that the Section will be eminently successful. We ask for it the co-operation of all who have the best interests of our profession at heart.

A circular will ere long be issued by the Executive Committee, giving status of the preparatory work for the Congress.

J. Taft,
of Section D. O. S.

---

### DR. R. L. ROBBINS.

At a meeting of the Trustees of the Boston Dental College, held January 6th, the following resolutions were adopted, viz:

Whereas, God in His inscrutable wisdom has removed from a sphere of usefulness in this life, to a life hereafter, Dr. R. L. Robbins, therefore,—

*Resolved,* That the members of the Board of Trustees of the Boston Dental College recognize in Dr. Robbins an efficient and much devoted member of this Board from its organization to the time of his death.

*Resolved,* That the Trustees of the College deplore the loss of a member so prominent, useful and devoted to the best interest of the College.

*Resolved,* That the Trustees feel that they have not only lost a very valuable co-worker in the interest of the College, but a friend whose wise counsel was highly esteemed. His most marked characteristics were his strict integrity and conscientiousness, never admiting or tacitly acquiescing in any act tainted with dishonor.

*Resolved,* That the members of this Board extend their heartfelt sympathies to the bereaved family, with the assurance of their high appreciation of his long, faithful and active service for the best interest of the College.

*Resolved,* That these resolutions be entered upon the records of the College and a copy sent to his bereaved family.

I. AYLING,
GEO. B. HARRIMAN,
J. M. DALY,
W. P. LEAVITT,
Committee on Resolutions.

CHAS. H. OSGOOD, Secretary.

---

### DENTAL SOCIETIES.

What the average dentist most needs is to be brought into contact with the leading and successful men of his profession; with those of whom he has often read and heard. His mind is thus led into new channels, and he gets broader views of his chosen work. The greatest benefit to be derived from dental societies is the elevating influence which they exert. They are not so much teachers of dentistry as educators in the broadest sense of the term. The ideas gained from reading alone are at best but superficial. To spur men on to a practical application of their readings we need personal contact with the men who stand as the exemplars. Then we learn that they are but human. The glamour that surrounds them when we know them only by their writings is removed, and we see that it is quite within our own power to reach their level and to practice by their standard.

R. I. PEARSON.

---

### MENDING RUBBER DAM.

Every dentist has been more or less troubled by cutting the rubber dam after being placed in the mouth. I have a way of repairing the break which I think may be of interest to the readers of the INDEPENDENT PRACTITIONER.

As soon as the rubber is torn place a napkin under it to stop the flow of saliva, and wipe it dry. Moisten the surface of a piece of vulcanizable rubber with chloroform, place it over the break and hold it in position a moment, when it will be found firmly adherent. I send you a piece of dam thus mended, that withstood an operation lasting three hours. F. A. GREENE.

This is an excellent and practicable suggestion, one that we have personally tried and approve. A. M. D.

---

### KANSAS STATE DENTAL ASSOCIATION.

The fifteenth annual meeting of the Kansas State Dental Association will convene at Topeka, on Tuesday, May 4th, continuing three days. This meeting will be made the most interesting and profitable one in the history of the Association. Members of the profession in other States are cordially invited to be present. Topeka is easy of access and has excellent hotel accommodations.

C. B. REED,
Secretary, Topeka, Kans.

### MARRIED.

In Newport, R. I., February 3, Charles Albert Brackett, D. M. D., Professor of Dental Pathology and Therapeutics in Harvard University, and Mary Irish Spencer, of Newport.

May this new demestic hearthstone, so happily laid, prove a haven of rest and peace, and may Prof. Brackett's matrimonial bark aye sail quiet seas, wafted by none but favoring gales.

---

### LOUISIANA STATE DENTAL SOCIETY.

This society will hold its next annual meeting in Tulane Hall, at New Orleans, March 4th, 5th and 6th, 1886. The Mardi Gras festivities, the Grand Exposition, with cheap railroad rates, should secure a large attendance. All will be welcome.

P. J. Friedrichs,
Chairman Executive Committee.

---

Professor Huxley's Table gives the weight of a well-proportioned man as 154 pounds, distributed as follows: Muscles, tendons, etc., 68 pounds; bony skeleton, 24 pounds; integument, 10½ pounds; fat, 28 pounds; brain, 3 pounds; viscera of thorax, 3½ pounds; abdominal viscera, 11 pounds; blood, 7 pounds.

Such a man should consume each day, beefsteak, 5,000 grains; bread, 6,000 grains; milk, 7,000 grains; potatoes, 3,000 grains; butter, 600 grains; water, 22,900 grains. His heart should beat 75 times per minute; he should breathe 15 times per minute. In every twenty-four hours he would vitiate 1,750 cubic feet of air to the extent of one per cent. He should throw off by the skin 18 ounces of water, 400 grains of solid matter, and 400 grains of carbonic acid every twenty-four hours, the total loss in that period of time amounting to six pounds of water and over two pounds of other matter.

---

The Ohio State Journal publishes an article bearing distinguishable earmarks, the aim of which is to combat the opinions advanced in a paper read last year before the American Dental Association, that the earthy phosphates are never directly assimilated as tissue food when administered for that purpose.

The writer is either obtuse or disingenuous. He fails to comprehend the point, or he is endeavoring to set up a man of straw for the purpose of showing how easily he can demolish it. The paper read at Minneapolis condemns the giving of earthy phosphates for *trophic* purposes, but asserts that they may, and do in some instances, act remedially. The subject was physiology, and not pathology. There is a difference between a medicine and a food.

---

A Plaster Cast of a nose, or any external organ which has deep undercuts, may be made as follows: Melt paraffine in a water bath, and with a soft brush paint the tissue over, laying on the first coat very quickly. Continue to add paraffine until a coating an eighth of an inch thick is obtained. This can be separated by cutting at the necessary points, and again placing the parts in contact, when the plaster cast may be made, pouring the batter in and out two or three times, to avoid air bubbles.

Children are particularly prone to disorders of the digestive apparatus, and the most prevalent form is indigestion, especially during the summer, owing to the extreme heat and the ingestion of food entirely unsuited to the age of the child. Very frequently, on questioning the mother as to whether she had given anything but the breast to an infant of a few months, I have been informed that she gave it a taste of meat, potato, or some other article which it is unable to assimilate. It seems entirely beyond her comprehension that she is giving a poison to the child, and is slowly starving it, from the fact that the absorbents are unable to take it up to supply the wants of the economy.—*The Polyclinic.*

---

Apropos to the Article by Dr. Spaulding in the last number of this journal, L. D. Caulk states that for the past seven years he has sold large quantities of his cements in the German markets; that for five years the demand for his "Par Excellence Alloy," which contains a percentage of copper, has been very large in fifteen foreign countries, and that the demand for them is constantly on the increase abroad. He says that a large majority of those who use cements do not know how to mix them properly.

---

The Dental Profession may consider itself greatly indebted to Dr. J. L. Williams, of Philadelphia. He has made decided improvements in porcelain teeth for crowns and bridge work, which, at a recent meeting of the First District Dental Society, he formally presented to the profession with an unfettered privilege to use them, although the doctor has refused tempting offers for exclusive rights to his improvements, from several parties. Such magnanimity should be widely recorded.

C E. F.

---

There is a desire on the part of some to have the next session of the American Dental Association meet in San Francisco. An excellent opportunity is offered, and nominal, or certainly phenomenal, railroad rates may be secured, the same that are offered the Grand Army of the Republic, which meets on the Pacific Coast. Will all who approve of this place of meeting please address

A. M. Dudley, Salem, Mass.

---

Dr. E. Parmly Brown says that bridge work is called new, but that it is at least 2,000 years old, a specimen from a grave in Etruria being now in existence.

Yes, and Dr. Brown gives the credit for the first presentation of the fact to the Independent Practitioner, which is more than is done by *Caulk's Annual*, from which the above was taken.

---

The firm of A. W. See & Co. has been organized to deal in dental goods. Their place of business will be 1347 Broadway, New York, and the firm will be composed of A. W. See and Mr. Fagin, recently in the employ of R. S. Williams, and E. A. Pierce, for twenty-three years with the S. S. White depot at Boston. The new firm will be the New York agents of Johnson & Lund.

---

The Odontological Society of Western Pennsylvania will hold its next quarterly meeting at the office of Dr. J. G. Templeton, 509 Penn Ave., Pittsburgh, on Tuesday evening, March 9th.

AN AMERICAN DENTIST, located for some years in a first-class European city, desires to sell his practice and return to this country, where he wishes to spend his declining years. He has a first-class clientele, and will give to the right man exceptional chances. His address will be furnished by the editor of this journal.

---

DIALOGUE BETWEEN TWO PHYSICIANS.—Dr. A——. Are you in general practice, or do you confine yourself to a specialty? Dr. B——. Oh, I am a specialist. Dr. A——. And your field is—? Dr. B——. My field is bounded above by the diaphragm, and below by Poupart's ligament.

---

THE FIRST DISTRICT DENTAL SOCIETY, of New York numbers about ninety active members. The meetings and clinics are largely attended, and are a source of great interest. Usually from one hundred to one hundred and fifty dentists are present on these occasions. X.

---

DR. S. S. SOUTHWORTH, of Sacramento, Cal., formerly of Niagara Falls, N. Y., and Brockville, Ont., is one of the State Board of Dental Examiners for California.

---

THE BRITISH DENTAL ASSOCIATION has been in existence but six years, and has six hundred members. It is the governing body of the dentists of England.

---

THE MEDICAL RECORD estimates the whole number of physicians in the United States, regular and irregular, active, passive and neuter, at 120,000.

---

DR. A. W. HARLAN was duly dined and wined by a party of his friends, at Donovan's, in New York, on his return from his European trip.

---

DR. O. W. HOLMES says that charlatanism always hobbles on two crutches—the tattle of women and the certificates of clergymen.

---

TWENTY FIVE DEATHS occurred in London last year from hydrophobia. The average number for the preceding ten years was six.

---

THE MICHIGAN STATE DENTAL ASSOCIATION meets at Ann Arbor on Tuesday, March 16th, and will continue in session four days.

---

DR. C. W. SPALDING, of St. Louis, is spending the winter in New Mexico, where he is engaged in mining enterprises.

---

DR. S. G. STEVENS, formerly of Lynn, Mass., has removed to Boston, and opened an office at 175 Tremont Street.

---

WM. H. VANDERBILT'S medical bequests were $100,000 to St. Luke's Hospital, and $50,000 to the Home for Incurables.

---

THE PARIS ADDRESS of Dr. G. C. Daboll, is for the present, "Care of American Exchange."

---

THE CHICAGO DENTAL COLLEGE has organized a Spring course of lectures.

